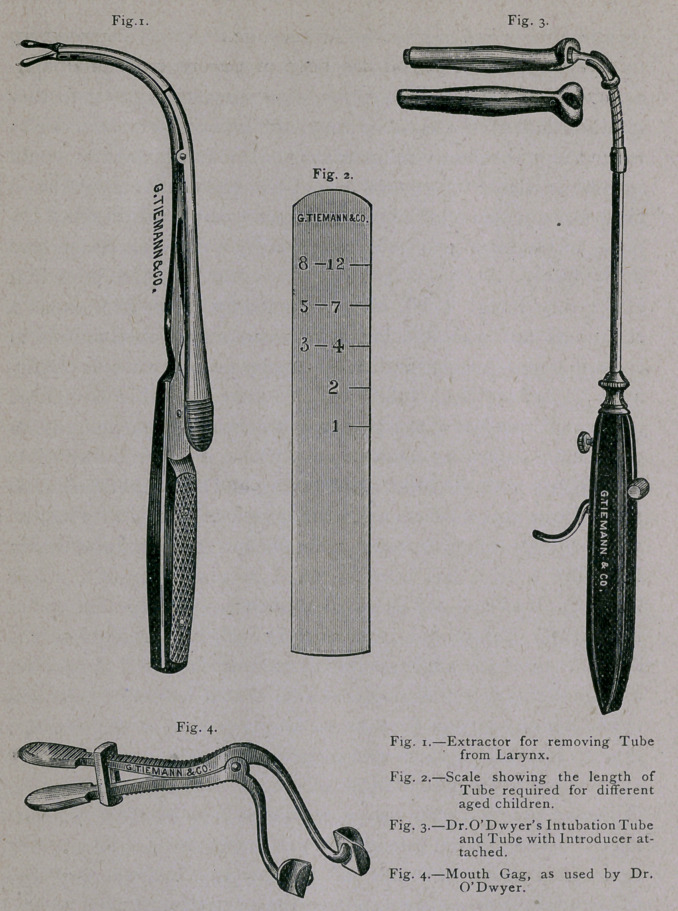# Intubation of the Larynx*From a paper on Diphtheria, read before the Buffalo Medical Club, June 9, 1886.

**Published:** 1886-07

**Authors:** A. R. Davidson

**Affiliations:** Professor of Medical Chemistry and Toxicology, Medical Department Niagara University, one of the Physicians to the Buffalo Hospital of the Sisters of Charity, etc.; 369 Pearl Street


### *Intubation of the Larynx.

By A. R. Davidson, M. D.,

Professor of Medical Chemistry and Toxicology, Medical Department Niagara University, one of the Physicians to the Buffalo Hospital of the Sisters of Charity, etc.

It is to this stage of the disease, to a discussion of the resources of our art, at a time when "hope is unwilling to be fed," that I would now invite your attention. A child is struggling for breath; at each attempt at inspiration there is a depression of the sub-thoracic tissue, and even of the soft tissues above the sternum. The respirations are frequent and stridulous, pulse small and frequent, the eyes listless and pupils dilated, mucous membranes are blue, the vesicular murmur is faint or absent. You know that the diphtheritic membrane is occluding the larynx. You know, too, that relief is possible only by the removal of the mechanical obstruction in the air tract. You have probably proposed tracheotomy to the parents some hours before, and have met with a refusal to permit it, with the assertion that they have known of several children who had their throats cut, and died, and if the baby must die, they will not allow it to be tortured. And, as you remember, perhaps, not a few cases upon which you have operated, and death followed, and Jacobi's statement after an experience of nearly two hundred operations, that he operates, only to avoid the distressing scene of death by suffocation, you probably feel that you have done your entire duty by suggesting it, and that you are not called on to insist upon, or even to urge it.

* From a paper on Diphtheria, read before the Buffalo Medical Club, June 9, 1886.

If now in the extremity of their distress, the parents consent and you do operate. You will, in perhaps nine cases out of ten, find that after the death of the child, the parents will, in their hearts believe, and oftentimes with their mouth confess, that the child would have got well if it had not had its throat cut.

Do not understand me as condemning tracheotomy. While I look upon it as the most unfavorable of all operations, if it does no more good than to furnish a few hours of comparative relief from suffering, it ought to be performed, if there is no other recourse. It is, however, the custom of the advocates of tracheotomy, to argue that the mortality is so great, because the operation is postponed until too late, and they advocate opening the trachea before there is any immediate risk of life. The operators who make any favorable showing, are those who act upon this principle; but I question whether the vast majority of such cases would not have recovered without an operation, indeed, rather, whether they did not get well in spite of it. Early operations are defended chiefly on the ground that the operation itself is not a serious one.

In diphtheria I believe it is always a dangerous one. It is now generally accepted that the membranes in the throat should not be interfered with for fear of provoking fresh inflammation and formation of membranes, and it is unreasonable to suppose that the opening of the trachea in a diphtheritic child does not add an extra risk. As a matter of fact, we know that the wound often takes on a diphtheritic inflammation. The irritation of the tube and the sensitiveness of the mucous membrane to changes of temperature, causes always a tracheitis, inducive rather to an extension of the membrane than the contrary. As Cohen puts it, "The casualties which prevent recovery after tracheotomy for diphtheria, arise from two sources. Those of one set are incident to the disease, and those of the other *to the operation.* The first will be merely mentioned. They comprise extensions of the pseudo-membrane, systemic infections, paralysis, nephritis, pneumonia and heart-clot. The accidents of the second class comprise hemorrhage, (primary and secondary,) emphysema, inflammation of the track of the incision, erysipelas, abscess, gangrene, diphtheria of the wound, ulceration of the trachea and pneumonia."

Before we subject our patients to the dangers involved in an operation, we must be sure that the risks entailed are more than compensated for by advantages gained—*e. g.* that the formation of membranes is arrested by an early operation. In my opinion, this has not been demonstrated, and therefore the operation of opening the trachea should be postponed to the latest possible minute. With this statement some of you may disagree, but I suppose there is no division of opinion as to the propriety of advising it as a *dernier resort.* At the same time I doubt if there is one of us who is at all enthusiastic over it, or who would not gladly accept any substitute which offered a reasonable hope of relief.

To Dr. Joseph O'Dwyer, of New York city, belongs the great credit of originating and practising the method now known as *Intubation of the Larynx*, and which, we believe, will take rank with the great advances in this age of medical discoveries. The boldness, ingenuity and patient experiment and research which Dr. O'Dwyer has shown, is, perhaps, not more remarkable than the modesty and carefulness with which he has presented his achievements to the profession.

As far as I know, the first notice which appeared in the journals was a paper written by Dr. Brush, President of the Westchester County Society, and published in the *Medical Record*, Feb. 21, 1885, in which he gives an account of three cases of intubation by Dr. O'Dwyer, which he had witnessed in the New York Foundling Asylum. That the writer was enthusiastic in his expectations was apparent; but at the conclusion of his paper he says: "Dr. O'Dwyer has been working on this subject for more than two years, but feels that he has not yet attained that pitch of perfection which he hopes some day to achieve. Therefore, I do not feel at liberty to describe the instruments he uses for inserting or withdrawing the tubes he employs, neither have I thought it right to ask him for his other cases which he intends to give to the profession when has has perfected his instruments, and achieved that success which I am confident he will gain."

Progressive Chicago, however, could not allow the babies of New York to have any advantages not enjoyed by themselves, and in a very excellent article on the treatment of croup (Chicago *Medical Journal and Examiner*, June, 1885), F. E. Waxham, Professor of Diseases of Children, College of Physicians and Surgeons, reports one case of intubation. From his remarks, we are led to believe that the instruments had just been received when a case of croup presented itself requiring their use. Tubage was effected, with great relief to the patient—a child under three—but death ensued within thirty hours, and the case was reported within three hours subsequently in a paper before the Chicago Medical Society, an example to put slow Buffalo doctors to the blush.

In the *New York Medical Journal* of August 8, 1885, Dr. O'Dwyer publishes his first communication on the subject — but says, "It was not my intention to publish anything in relation to my method of tubing the larynx in croup, and kindred diseases, until I had brought my instruments to a greater degree of perfection than I can claim for them, at present." Already, however, others had written upon them, and it was necessary for him to make his own presentation of the subject. This he does in an interesting way, giving a short history of Bouchut's similar attempt and failure in 1858, and then recounts some of the difficulties he had met with and modifications of instruments employed during nearly five years of experiments. He describes his improved tubes, and says: "That in the few cases I have used the modification it has proved self-retaining, and should it continue to do so on a more extended trial, I believe there will be little scope for further improvement." But with a commendable frankness and caution, he says again: "As all my work, up to a very recent period has been of purely experimental character, and as I am not prepared, even now, to say that further modification of these tubes may not be necessary in order to make them absolutely self-retaining, without which they would not be available for general use, I will leave all consideration as to value of this method of treating croup and other acute stenoses of the larynx to be determined by a more extended trial in the future. At the same time I will venture the prediction that in the near future it will be recognized by the profession as a legitimate and valuable means of overcoming obstructions in the upper air passages, with a rapidity by no other means obtainable. I believe these tubes will also prove valuable as dilators in chronic stenoses of the larynx or trachea, and particularly in those cases following tracheotomy, where it is found imposible to dispense with the tube. Not having had any experience with such, I can give no facts as to the length of time a tube can be worn without injury, but some valuable inferences may be drawn from the time it has been retained in acute stenoses. For instance, in two of my cases of diphtheritic croup that ended in recovery, the cannula was worn in each for the space of ten days, without the slightest impairment of the vocal apparatus, and from this it is reasonable to infer that it would be tolerated in the healthy larynx for a much longer period, and probably, if worn intermittingly, for an indefinite period."

The moderate anticipations of success thus expressed by Dr. O'Dwyer nearly a year ago, seem already to be more than realized. The general introduction of intubation has been much retarded by the difficulty of getting the instruments, Dr. O'Dwyer being unwilling to have them introduced until he had perfected them as far as his experience would allow. I have the pleasure of showing you, through the kindness of the Messrs. Tieman & Co., the latest modification of the instruments, as now commended by Dr. O'Dwyer. Tieman writes me that they have more orders than they can at present fill.

Fig. 1.

Fig. 2.

Fig. 3.

Fig. 4.

Fig. 1.—Extractor for removing Tube from Larynx.

Fig. 2.—Scale showing the length of Tube required for different aged children.

Fig. 3.—Dr. O'Dwyer's Intubation Tube and Tube with Introducer attached.

Fig. 4.—Mouth Gag, as used by Dr. O'Dwyer.

The set of instruments consists of five laryngeal tubes with obturators, an applicator, an extractor, a gauge and a gag. The tubes vary in length from 1¾ to 2½ inches, suitable for cases from a few months to ten or twelve years of age. They are of metal, plated with gold. When inserted, the entire tube is within the larynx and trachea; the upper end, resting upon the ventricular bands, is supposed not to interfere with the functions of the epiglottis; but, as Dr. O'Dwyer points out, the epiglottis is only an accessory to the closure of the larynx, and the other more important factor, the action of its constrictor muscles is presented by the presence of the cannula, it is evident that the deglutition of fluids can never be perfect with a tube in the glottis. The lower end of the tube is about half an inch from the bifurcation of the trachea. The swell of the tube, from the neck to a little above the centre, is designed to prevent its expulsion by coughing or expectoration. Each tube has, at its upper extremity, an eye for a silk thread, by which the operator can quickly remove it if not properly introduced. Each tube is provided with an obturator, which serves the double purpose of being the point of attachment for the applicator, and also giving to the extremity of the cannula a rounded and smooth surface. The applicator is shown in the cut with the tube attached. By means of a thumb-piece upon the handle of this instrument, the tube can be held in place while the obturator is withdrawn. The instrument for removing the tube has a jointed point, which, after insertion into the orifice of the tube, can be made to expand, giving a firm hold upon the tube. The gauge is designed to determine the length of tube to be used for any given age.

In performing the operation, Dr. Ingalls gives the following suggestion: The child should be wrapped in a sheet or shawl, which will pinion the arms, and then held upright in the nurse's lap; an assistant holds the child's head. The gag is then introduced between the jaws, and opened as wide as need be, but not with great force. Dr. O'Dwyer says that it is unnecessary to use the gag with infants who have not back teeth. The physician, sitting in front of the patient, passes his left index finger over the base of the tongue and down behind the epiglottis, and with it guides the end of the tube into the glottis. The handle of the applicator should be held near the child's sternum until the end of the tube has reached the pharyngeal wall, when the handle is rapidly elevated, and tube directed downward and forward along the index finger into the larynx. This will not be found difficult, but the infant's epiglottis is so small and flaccid, that the operator may not be able to recognize it, though he will have no difficulty in recognizing the larynx as a whole, which, except that it is slightly irregular, feels much like the end of one's little finger. The operator should not expect to detect the opening of the glottis, but must be guided by his anatomical knowledge to pass the tube into the center of the larynx. Unless he is careful to carry the handle of his instrument high, and thus bring the tube as far forward towards the base of the tongue as possible, the tube will pass into the œsophagus. While it is desirable to accomplish this portion of the operation as quickly as possible, it should not be done with too great haste. Ten or twenty seconds, which is a long time for this portion of the operation, may be taken without danger. If the tube is not then introduced, it should be removed for a minute or two, to allow the child to breathe, and then the operation may be repeated; but if the tube seems to be in the proper position, whether the operator is certain of it or not, the slide upon the handle should be crowded forward, so as to disengage the obturator, which is then withdrawn. Some cough will occur at once, and if the tube has not been inserted into the larynx, or if it has not been passed down so that the rim rests on the vocal cords, it is likely to be expelled, and may be seen or felt in the back part of the mouth. If the tube has been properly inserted, respiration will become easier, and after a few minutes the operator cuts one end of the silk thread, passes his finger behind the epiglottis, and holds the tube while the thread is withdrawn.

It has been demonstrated that the constant irritation produced by the contact of the thread with the epiglottis and base of the tongue, is in some cases unendurable, and also it is difficult to prevent the child from pulling at it; therefore, the thread is always to be removed. The removal of the tube is more difficult than its introduction, according to the experience of all the operators, it being no easy task, with a struggling child, to guide the extracting instrument into the narrow aperture of the tube, and in many cases an anæsthetic is needed.

The advantages which intubation possesses over tracheotomy are thus summarized by Dr. Waxham, who has had by far the largest number of operations up to the present time:

1. No opposition is met with on the part of parents—quite a contrast with the difficulty which we usually meet with in obtaining the consent to tracheotomy.

2. It relieves the urgent dyspnœa as promptly and as effectually as tracheotomy, and if the child dies, there is no regret that the operation was performed, and no discredit attached to the physician.

3. There is less irritation from the laryngeal tube than from the tracheal cannula. As the tube is considerably smaller than the trachea, it does not press upon it firmly at any portion, excepting at the chink of the glottis.

4. Expectoration occurs more readily than through the tracheal tube.

5. As the tube terminates in the throat, the air that enters the lungs is warm and moist from its course through the upper air passages, and there is less danger of pneumonia.

6. It is a bloodless operation.

7. It is more quickly performed, and with less danger.

8. There is no open wound, which may be the source of constitutional infection.

9. Convalescence is more rapid, and there is no ghastly wound to heal by slow granulations.

10. The patient does not require the unremitting care of the surgeon as in tracheotomy.

11. I believe it to be a more successful method of treating croup, either diphtheritic or membraneous, than tracheotomy.

The above list gives the advantages of intubation, but let us consider, too, the objections:

1. The difficulty of inserting the tube. This, though admitted, is certainly less than tracheotomy.

2. That the tube may become blocked with mucus or membrane. The recorded experience in thirty-seven cases would indicate that this does not occur, because—and this is one of the marked advantages of tubage over tracheotomy, the patient has the ability to compress the air in the lungs and expel it with an explosive force; in other words, to cough—thereby clearing the tube.

3. That the tube may slip through into the trachea. If too small a tube is used, this may happen, but from the length of the tube, it cannot sink out of reach, and may be removed by the mouth, or by tracheotomy.

4. That the child cannot swallow well. This is true only of fluids, and it is necessary to avoid giving liquids by the mouth. A few drops will trickle into the trachea and cause violent coughing, and this irritation will often lead to pneumonia. Dr. Waxham has devised a feeding-bottle for young infants; and it may be necessary to use a small-sized œsophageal tube in some cases.

5. The cannula may be coughed out in the absence of the physician, and death ensues before he can be summoned to re-introduce it. This danger is not nearly so great as that which attends the wearing of the tracheal tube.

The only discouraging report which I find, is in the report of an unsuccessful case by Dr. Hatfield, Professor of Diseases of Children, in Chicago Medical College. Although he called to his aid the skill and experience of *Waxham*, he reports that it required six efforts to insert the tube before successful, and when at last accomplished, it seemed, for the first few moments, that the child would die of asphyxia. Nevertheless, it safely struggled through this, its breathing became easy, and the child dropped into a tranquil sleep. In twenty-four hours, the dyspnœa was as painful as before. As a forlorn hope, the tube was removed, in the hope of cleaning the trachea of mucus and disintegrated membrane. The lumen of the tube was found to be free; but œdema of the parts, and the critical condition of the child, made all efforts to again introduce the tube futile, and "the child died from accumulation of mucus in the lungs, twenty-seven hours after the first introduction of the tube." Dr. Hatfield says (it is to be remembered that he wrote in July, 1885), that all of the cases which had come to his knowledge had terminated fatally, and asks for other than æsthetic reasons for practicing intubation. He says, the serious and grave objections to the tubes are the difficulty of introduction, and that even when properly introduced, their presence does not insure permanent relief.

Since that time, however, the statistics of the operations far surpass, in percentage of recoveries, the result obtained by tracheotomy, and intubation gives as good promise of permanent relief. The extension of the diptheritic process into the bronchial tubes is rather more probable after tracheotomy.

In conclusion, gentlemen, I would express the opinion that tubage of the larynx bids fair to take a high place among the agencies of our art to relieve suffering and to save life. It is too soon to pronounce absolutely upon its merits, but we have sufficient evidence of its success to entitle it to a trial in every case in which our other only recourse would be tracheotomy, and it is probable that within a short time the discussion will be not as to its utility, but as to how early, in acute stenosis of the larynx, the glottis should be tubed.

369 Pearl Street.

## Figures and Tables

**Fig. 1. Fig. 2. Fig. 3. Fig. 4. f1:**